# Accuracy of Patient-Specific Implants in Virtually Planned Segmental Le Fort I Osteotomies

**DOI:** 10.3390/jcm12186038

**Published:** 2023-09-18

**Authors:** Reinald Kuehle, Mats Scheurer, Frederic Bouffleur, Jennifer Fuchs, Michael Engel, Jürgen Hoffmann, Christian Freudlsperger

**Affiliations:** Department of Oral and Maxillofacial Surgery, University of Heidelberg, Im Neuenheimer Feld 400, D-69120 Heidelberg, Germany

**Keywords:** orthognathic surgery, Le Fort I osteotomy, segmental osteotomy, cohort studies, spiral cone beam computed tomography, maxilla, osteotomy

## Abstract

(1) Background: In orthognathic surgery, segmental Le Fort I osteotomies are a valuable method to correct maxillary deformities or transversal discrepancies. However, these procedures are technically challenging, and osteosynthesis can be prone to error. (2) Methods: In this retrospective, monocentric cohort study, patients were enrolled who underwent a virtually planned segmental maxillary osteotomy during their combined treatment. Positioning and osteosynthesis were achieved by either a 3D-printed splint and conventional miniplates or patient-specific implants (PSI). The preoperative CT data, virtual planning data, and postoperative CBCT data were segmented. The deviation of all the segments from the desired virtually planned position was measured using the analysis function of IPS CaseDesigner. (3) Results: 28 Patients in the PSI Group and 22 in the conventional groups were included. The PSI group showed significantly lower deviation from the planned position anteroposteriorly (−0.63 ± 1.62 mm vs. −1.3 ± 2.54 mm) and craniocaudally (−1.39 ± 1.59 mm vs. −2.7 ± 3.1 mm). For rotational deviations, the pitch (0.64 ± 2.59° vs. 2.91 ± 4.08°), as well as the inward rotation of the lateral segments, was positively influenced by PSI. (4). Conclusions: The presented data show that patient-specific osteosynthesis significantly reduces deviations from the preoperative plan in virtually planned cases. Transversal expansions and vertical positioning can be addressed better.

## 1. Introduction

Treating severe skeletal deformities and malocclusion by orthodontic treatment in combination with orthognathic surgery is well established, and surgical methods have been elaborated over the years. Of these, Le Fort osteotomies, in all their modifications, are some of the most frequently performed procedures in oral and maxillofacial surgery. The success and development of orthognathic surgery have historically been connected to technological innovations due to the high demand for precise positioning and osteosynthesis of bony segments [1]. Despite tremendous improvements in three-dimensional (3D) imaging due to the wide availability of cone beam computer tomography (CBCT) scans, transferring the exact treatment plan to the operative site remains challenging. Addressing this challenge, two technological innovations have influenced orthognathic surgery in recent years to a great extent: 3D virtual planning and additive manufacturing [2,3].

While virtual planning and the simulation of orthognathic procedures have changed the basic principles of planning, resulting in a shift from orthognathic to orthofacial surgery [2], additive manufacturing helped facilitate this. Additionally, virtual planning software solutions help to reduce the “black box phenomenon” of the operation by visualizing the postoperative changes to the patient. The correction of complex deformities can be assessed in ample ways, and the surgical outcome can be achieved with a higher probability and accuracy. Various computer-aided design and machining (CAD/CAM) techniques to produce operation splints have been utilized to conduct these surgical plans with high precision. Some of these techniques are CAM-milling, stereolithography (SLA), selective laser melting (SLM), fused deposition modeling (FDM), and digital light processing (DLP). Of these techniques, resin-based 3D printing (SLA and DLP), a cheap and precise method of splint creation, has helped catalyze this process to a great extent.

While conducting the virtual plan with 3D-printed splints, osteosynthesis in the operating room is usually based on conventional mini plates and screws. However, hand-bent osteosynthesis plates can negatively influence the implementation of a virtual plan [4,5]. With the possibility of registration errors, possible joint dislocations, and user-based errors, virtually planned but splint-based surgery can lead to unwanted segmental displacements [6,7]. Patient-specific osteosynthesis suggests reducing the displacement by fitting in a specific position and guiding the surgeon to position the bony segments through drilling and cutting guides. Selective laser melting of titanium is the most common technique to manufacture individualized osteosynthesis, yet CAD/CAM machining and preoperative individualization of conventional plates have been described.

The surgical procedure of a multisegmented maxillary osteotomy was already described by Steinhauser in 1972 and Turvey in 1985 as an intraoperative alternative to two-stage surgical palatal expansion (SARPE). The procedure’s safety has been described in several smaller case series [8,9]. If conducted adequately, the maxillary multipartition causes hardly any additional stress for the patient. When indicating segmental Le Fort I osteotomies, the possibility of palatal perforation of the mucosa and an increased risk of tooth damage due to inter-radicular osteotomies should be considered. Segmental maxillary osteotomies are frequently used to correct pronounced skeletal discrepancies, such as relevant differences in the Curve of Spee, transversal deficits, or a frontal open bite.

**Figure 1:**
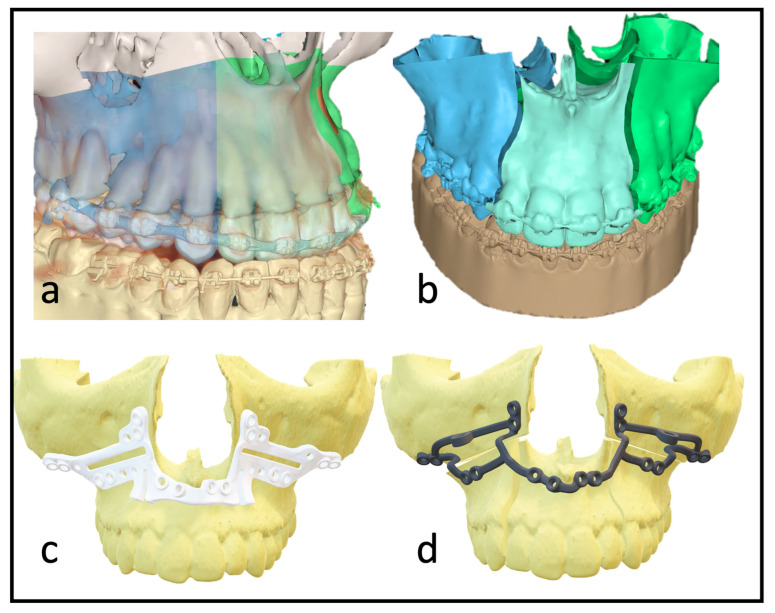
(**a**) Depiction of the roots to indicate the ideal plane for the osteotomy. (**b**) Virtually planned “three-piece maxilla” to compensate for transversal deficit and close the anterior open bite. (**c**) Marking and drilling guide: slots mark the osteotomy between canines and lateral incisors. The smaller drilling holes are for the fixation of the guide; the larger holes will hold a metal sleeve to guide the holes for the PSI fixation. (**d**) 3D design of the patient-specific implant with cranial stabilization bars, which can later be removed.

Previous studies have rarely shown increased risks of complications with additional maxillary multipartition compared to standard Le Fort I osteotomy [10]. The procedure can eliminate the burden of a preceding surgery (SARPE) if the indication is given, and the transversal development is moderate. The indication for maxillary multipartition is mainly found in transversal deficits up to 6–7 mm. In rare cases, correcting the transversal ratios or asymmetry of the alveolar crest may be necessary even after SARPE. In segmental maxillary osteotomies, a distinction is made between the two-piece maxilla (medial split), three-piece maxilla (Y-cut or H-cut), and asymmetrical modifications. Performing these osteotomies requires more extensive preoperative planning (Figure 1) and a thorough surgical approach that is often more time-consuming in theatre. Additional osteosynthesis and measures, such as splints with wiring, are required to increase stability. As the risk of transversal collapse is always given in segmented maxillary osteotomies, prolongated splint use with palatal extensions or wiring has been described and widely used.

With multisegmented maxillae, the patient requires longer retention to ensure long-term stability, as there is always a risk of transversal collapse or instability. The rigidity of the patient-specific implant allows better position-finding and promises higher dimensional stability. Full arch designs can eliminate the need for additional osteosynthesis between segments (Figure 1d). The virtually designed implants are based on interconnected L-plates.

Following maxillary segmental surgery, the final orthodontic alignment of the dental arches can be conducted on an optimized dental arch. It is possible to shorten the duration of subsequent orthodontic therapy if the planned position of the tooth-bearing segments can be achieved. Consequently, surgical precision is of particular importance. 

Previous studies have shown the beneficial effect of patient-specific osteosynthesis in nonsegmental maxillary procedures in randomized controlled studies [5,7]. To the best of our knowledge, there are no 3D analyses of bone segments in multipiece maxillae based on the established analysis method that take the influence of different osteosynthesis techniques into account [11]. 

In this study, the use of patient-specific implants compared to conventional osteosynthesis in patients with segmental maxillary procedures is investigated in terms of accuracy. The semi-automated evaluation tool of the newest version of IPS CaseDesigner (KLS Martin, Tuttlingen, Germany) is evaluated and used. The group of authors hypothesizes (h0) that the virtual 3D treatment plan can be more precisely transferred to the operative site with the help of patient-specific implants.

## 2. Materials and Methods

### 2.1. Patients

The inclusion criteria for patients for this retrospective cohort study were:

(1) a history of bimaxillary orthognathic surgery using Le Fort I osteotomy, including two- or three-piece maxilla in the Department of Oral and Maxillofacial Surgery at the University of Heidelberg; (2) virtual surgical planning with IPS CaseDesigner; (3) either the use of additively manufactured, patient-specific implants from KLS Martin, Tuttlingen, Germany or solely 3D-printed interocclusal splints with miniplates (Modus 1.5 Orthognathic, Medartis, Basel, Switzerland); (4) sufficient pre- and postoperative 3D imaging CT/CBCT.

The exclusion criteria for patients for this retrospective cohort study were: (1) no virtual planning; (2) subjects with severe craniofacial asymmetries and deformities; and (3) a history of, or revision, surgery.

We screened our digital patient records (ISH, SAP, Walldorf, Germany) and the radiological database (PACS, Philips Medical Systems, Drachten, Nederland B.V.) to identify patients who met the inclusion criteria. The data of these patients were exported and anonymized. After anonymization, the segmentation process began.

### 2.2. Data Collection

**Figure 2:**
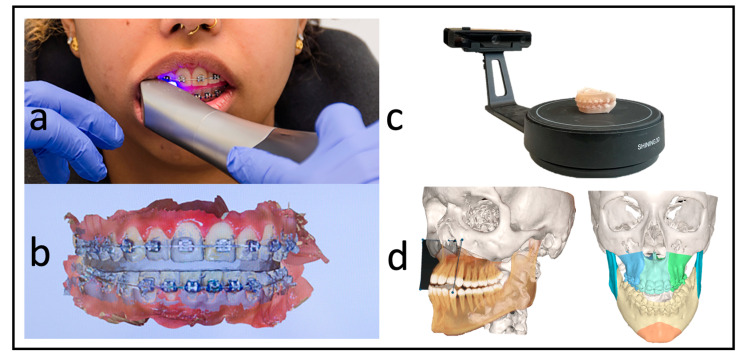
Workflow without dental impressions. (**a**) Intraoral scan with a wax bite. (**b**) Digital scan and registration. (**c**) Re-digitalization using a surface scanner. (**d**) Fused scan and CT for virtual surgical planning.

Each patient received a low-dose CT (Siemens Somatom Definition AS 64) scan of the skull before surgery (T1) and a CBCT scan approximately three days after surgery (T2) for postoperative control. We used the Orthophos SL (98 kV at 3 × 10^8^ mA pulsed mode, spherical volume of 15.4 cm, scan time of 14 s, isotopic voxel size of 0.25 mm, Sirona, Bensheim, Germany) for the postoperative images. The preparation of the surgical planning files per our department’s routine protocols was as described by Swennen [12]. In addition to standardized photo documentation to record the natural head position, intraoral scans as *.stl files (“standard triangle language”) (Primescan, Dentsply Sirona, Bensheim, Germany) and a wax bite to register the centric condylar position were also taken. After manual adjustment of the target occlusion using the 3D-printed patient models (Modelresin, Form 3B+, Formlabs GmbH, Berlin, Germany), these target occlusions were re-digitized back into an *.stl file using a model scanner (Shining 3D EinScan-SE, Hangzhou, China) (Figure 2).

### 2.3. Virtual Surgical Planning (VSP)

Digital treatment planning was performed using IPS CaseDesigner V. 2.4, a surgical planning and simulation software based on the individual patient datasets described below. The data included in the virtual planning were: (1) low-dose CT of the skull (DICOM dataset); (2) natural head position (NHP) standardized photos (*.jpeg); (3) intraoral scans (.stl); and (4) a digital model of the target occlusion (*.stl). These data were consecutively entered into IPS CaseDesigner V. 2.4 and merged to simulate the surgery based on the NHP. As part of this simulation, the planned osteotomy lines of the Le Fort-I osteotomy, as well as the maxillary split into the two- or the three-piece, were indicated. In the mandible, a sagittal split osteotomy was drawn and virtually performed. The desired target occlusion was defined by manual positioning with a 3D-printed model. After re-digitalization by surface scanning, the segments were placed in the planned position. In the case of bimaxillary procedures, a profile correction was made based on aesthetic facial features and clinical planning. Based on the resulting movements of the maxilla, conventional splints or the PSI with drilling and cutting guide were then fabricated. 

### 2.4. Operation and Techniques

**Figure 3:**
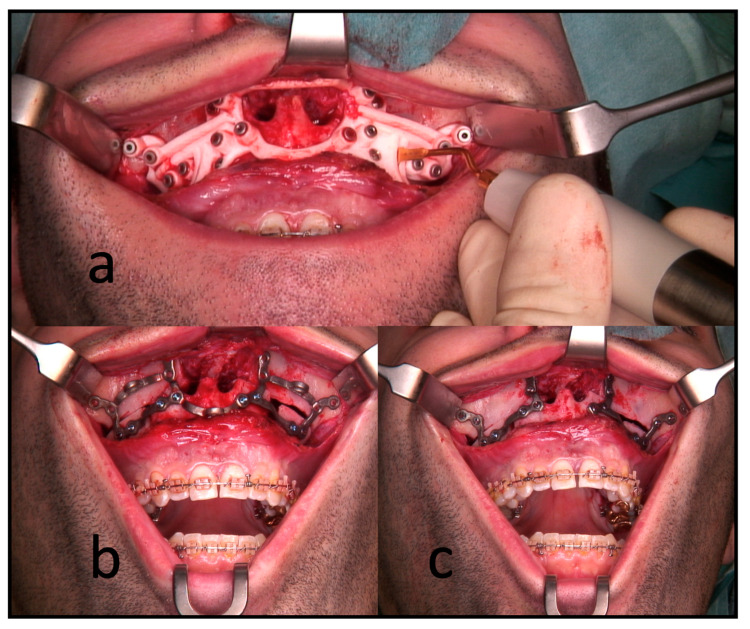
Intraoperative use of PSI for a three-piece maxilla. (**a**) Drilling and cutting guide: the piezo osteotome is used to conduct interradicular osteotomies. (**b**) The full arch patient-specific implant is used to position and osteosynthesize the maxillary segments without a surgical splint. Note the infraorbital and medial connection bars. (**c**) The connection bars are removed, leaving the patient with interconnected individual L-plates.

All the surgeries were performed by the authors, CF or RK, under general anesthesia. In the segmental maxillary osteotomies with PSI, the intraoral incision was extended from the first molar’s vestibulum to the other quadrant’s first molar. This was followed by subperiosteal preparation and denudation of the maxilla. Subsequently, the drilling and cutting guide was placed. After fixing this guide with 1.5 mm screws, the guided drilling was conducted by drilling along the metal tubes with a 1.1 mm drill for a 1.5 mm screw osteosynthesis. The horizontal osteotomy of the Le Fort I osteotomy and the inter-radicular osteotomies were conducted by piezo-osteotomy (Figure 3). Patient-specific implants fabricated by KLS Martin (KLS Martin, Tuttlingen, Germany) were used to position both the maxilla and the segments to each other with 1.5 mm screws without using a surgical splint. 

The conventional group used the same surgical approach; positioning was achieved with an intermediate splint and osteosynthesis by four L-plates (Medartis 1.5 Modus Orthognathic).

A postoperative CBCT scan was performed in both groups within the first postoperative week. Subsequently, this postoperative result was compared with the previous planning according to the following protocol.

### 2.5. Data Analysis

**Figure 4:**
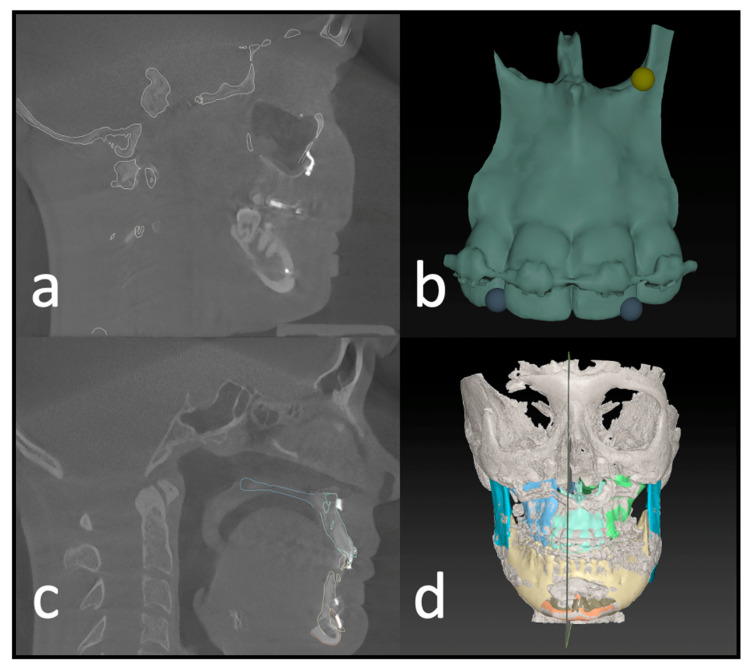
Postoperative analysis of a three-piece maxilla. (**a**) Matching of the preoperative CT and virtual plan to the postoperative CBCT scan. (**b**) Identifying landmarks on the segment that can be isolated on the postop CBCT. (**c**) Visual confirmation of the automatic matching process. (**d**) Overview of all segments matched to the postop imaging.

The initial preoperative VSP planning (T1) file was utilized to conduct the analysis, and the postoperative imaging (T2) was entered into the IPS CaseDesigner software V. 2.4 and saved as a separate file. The measurements in this study are based on the “compare analysis” tool, which is included in the newest version of IPS CaseDesigner. This semiautomated analysis tool is based on a two-step process. First, the pre- and postoperative imaging is segmented and matched as a CT on CT registration (CT to CBCT, in our case) as described by Maes et al. [13]. Reference for this is the unchanged part of the orbital, cranial, and skull base anatomy. The user identifies at least three points on the virtual anatomy; then, the automatic matching is validated by the user (Figure 4a). By matching the pre- to postoperative imaging, the virtually planned position of the segments is overlaid on the postoperative imaging. It is important to note that CT and CBCT scans can show the different quality of bone visualization. This possible error is overcome by visually confirming the segmentation of the bone on the CBCT with the outline of the segment created in the Virtual Surgical Planning software. If the segment aligns well, the analysis is continued. Otherwise, the threshold of the CBCT can be adapted, or the position of the virtual segment re-aligned. 

After identifying three anatomical points on each maxillary segment in the second step (Figure 4b), the software automatically calculates the deviation of each segment’s planned position. This deviation equals the translational movement that the automatic matching process (Iterative Closest Point (ICP) algorithm) determined while matching the postoperative position to the planned position of each segment. The theory behind the algorithm is an iterative reduction of the distances between two virtual objects as described by De Groeve et al. [14]. The matching result is then confirmed by the investigator (Figure 4c).

**Figure 5:**
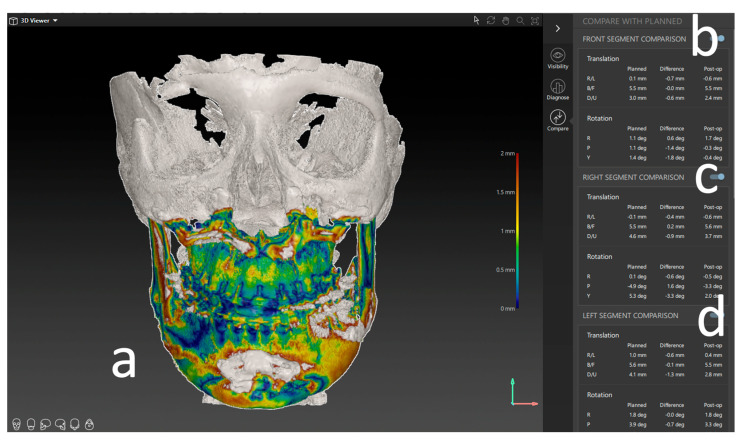
Results of the postoperative analysis of a three-piece maxilla. (**a**) Overlay and heatmap of the preoperative virtual plan and postoperative result in the form of a CBCT scan. (**b**) Deviation of the frontal segment. (**c**) Deviation of the right segment. (**d**) Deviation of the left segment. All measures are shown in lineal movement (mm) under translation and rotation (degree °).

The result of the ICP-based matching is a transformational matrix that describes the spatial difference between the two objects in XYZ linear and rotational measurements. These measurements are displayed as linear deviations in mm or degree for the specific segments (Figure 5).

### 2.6. Statistical Analysis

All the statistical analyses were performed using IBM SPSS software (version 25.0). Descriptive statistics for means and standard deviations for translation and rotation of the postoperative position compared to the planned position were calculated for both intervention groups (PSI, conventional splint). The Student’s *t*-test was used to compare the differences in accuracy between the two groups. *p*-values below 0.05 were considered statistically significant. There was no adaption for multiple testing due to this work’s descriptive and retrospective nature.

## 3. Results

### 3.1. Basic Characteristics

A total of 50 patients who underwent orthognathic surgery at the Department of Oral and Maxillofacial Surgery at Heidelberg University Hospital from 2017 to 2022 were recruited for this study.

Of these, 56% (*n* = 28) were operated on using patient-specific osteosynthesis; on 44% (*n* = 22), a conventional osteosynthesis and splint technique was used. Of both groups, *n* = 10 patients underwent a two-piece maxilla; the rest underwent a three-piece maxilla (*n* = 18 PSI; *n* = 12 splint).

The mean age at the time of the operation was 27.82 years, with an SD of 8 years. Of all the patients, 56% were female. The distribution of skeletal classes was balanced for a northern European country, with a dominance of 64% class II and 34% class III patients; only 2% were class I with an open bite.

### 3.2. Validation of the Method

The translational and rotational movements at a 95% confidence interval were analyzed. To address the validity of the method, 18 measurements were repeated by the main and a second investigator. From these measurements, the inter- and intra-rater consistency was calculated (ICC: inter/intraclass correlation). An excellent inter- (0.87) and intra-observer (0.83) correlation was found for the translational and rotational measurements. The intra-observer translational and rotational mean absolute differences (MAD) were 0.03 mm and 0.39°, respectively. The inter-observer MAD was 0.01 mm for translational and 0.37° for rotational movements. 

### 3.3. Results of the Measurements

The measurements quantify the difference from the virtual plan in six variables for each maxillary segment, three for linear movement: right/left, back/front, down/up in mm, and three for rotation: roll, yaw, and pitch. 

**Figure 6:**
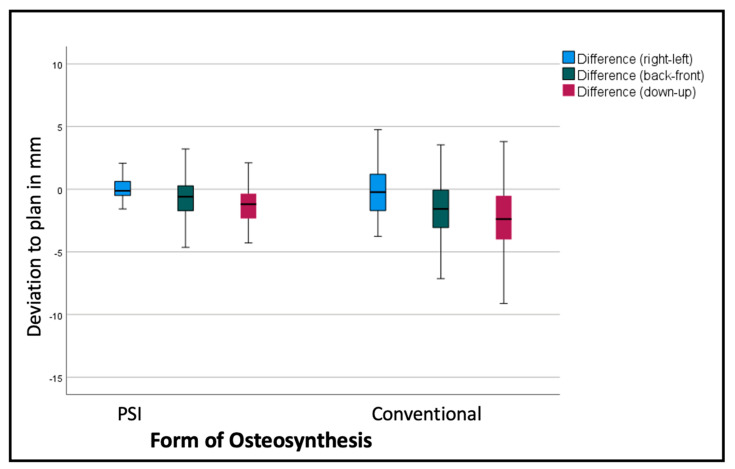
Deviation from the planned position of the segments (overall). Color code shows the different axes of movement in mm: blue (right−/left+); green (posterior−/anterior+); red (down−/up+).

Comparing the mean differences of these six variables for all the segments in both groups (PSI and conventional), the *t*-test showed significantly lower deviations in the PSI group: back/front (*p* = 0.035), down/up (*p* < 0.001), roll (*p* = 0.045), and pitch *p* < 0.001). The most substantial differences can be displayed for the linear measurements (Figure 6 and Table 1). For the transversal (right/left) deviations from the virtual plan, the absolute values are moderate in both groups, although the confidence interval (CI) is relevantly larger in the conventional group. For the other dimensions, the conventional osteosynthesis diverts more strongly from the plan (Figure 6). 

The segments are positioned by an average of −2.69 mm too low, with a standard deviation of over 3 mm, and too far back by 1.3 mm, with a standard deviation of 2.54 mm in the conventional group. The PSI segments are positioned by an average of 1.29 mm too low, with a standard deviation of 1.59 mm (*p* < 0.001), and 0.63 mm too far back, with a standard deviation of 1.62 mm (*p* < 0.001). Table 1 shows, more differentiated, the deviations of the single segments with larger differences in the lateral segments. 

**Figure 7:**
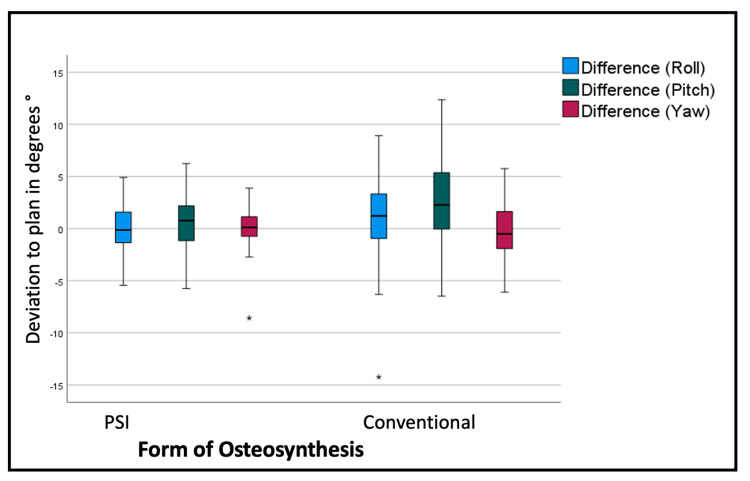
Deviation from the planned position of the segments (overall). Color code shows the different axis of movement in degrees (°): blue (roll+/−); green(pitch clockwise−/counterclockwise+); red(medial−/lateral+); * symbolizes outliers.

The overall assessment of the rotational deviation from the plan shows the most significant absolute values in the pitch rotation, i.e., a clockwise rotation from the lateral right-side perspective. The conventional osteosynthesis shows an overall pitch deviation of 2.9° with a standard deviation of 4.08°, in comparison to the PSI, with a variation of 0.64° with a standard deviation of 2.59° (*p* < 0.001). The segment-specific deviations from the VSP regarding rotation are displayed in Table 2 and Figure 7.

**Figure 8:**
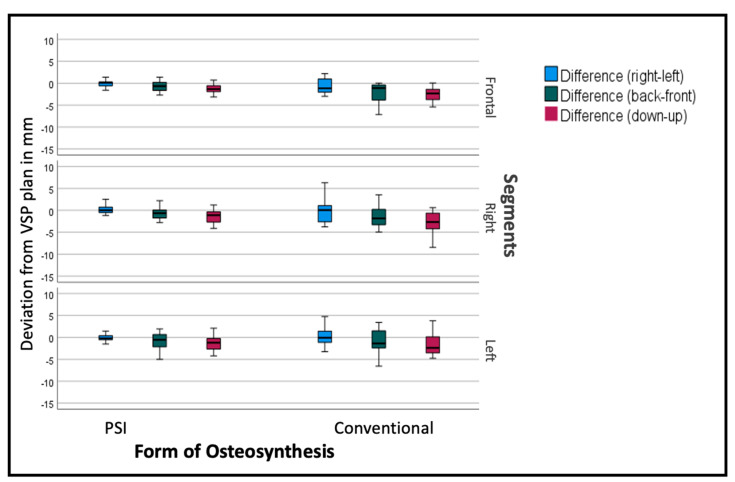
Deviation from the planned position of the segments (overall). Color code shows the different axis of movement in degrees (°): blue (roll+/−); green (pitch clockwise−/counterclockwise+); red (medial−/lateral+).

The deviation from the desired position was sub-stratified for the individual segments to display the effect of the PSI on each specific segment. As seen in Figure 8, the overall tendency can be seen in the individual segments as well, but to a different extent. The frontal segments show fewer problems concerning the vertical aspect. The lateral segments tend to have insufficient impaction, especially when conducted conventionally.

To assess the maxillary width development, the transversal movement of both lateral segments was added. All the medial movements were calculated as negative values, and all the lateral as positive values. 

Concerning the rotation of the lateral segments, a deviation from the plan to the medial aspect would be a negative value in degree. The rotation point for every segment was in the osteotomy between the incisors in a two-piece or between the canine and the second incisor in a three-piece maxillae.

**Figure 9:**
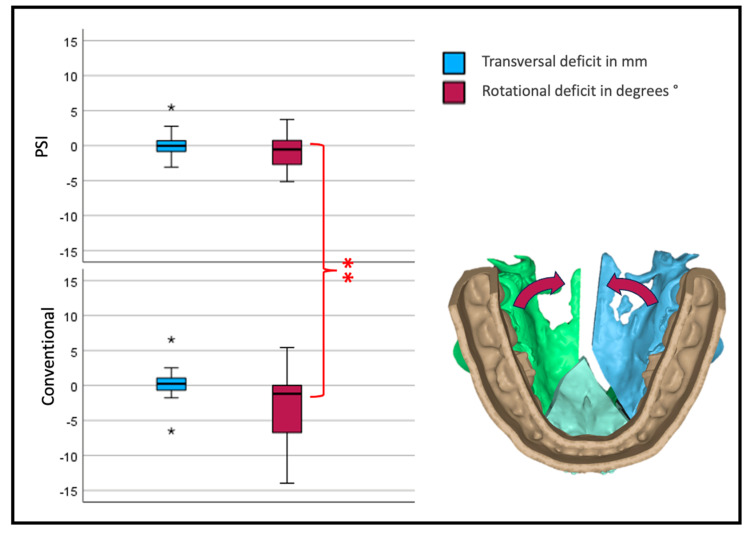
Transversal deviation from the virtual plan. Blue depicts translational; red, rotational deviation. The reference center for the analysis of the movement is in the anterior medial aspect of the segment. Therefore, transversal development is measured at the osteotomy anterior (three-piece) or medial (two-piece) of the lateral segments (* symbolizes outliers, ** symbolizes highly significant differences *p* < 0.001).

Interestingly, the transversal width shows no significant difference between the two forms of osteosynthesis. Looking at the yaw rotation, i.e., the rotation around the z-axis of the body’s absolute values, we see a significant and relevant inward rotation in the conventional group (*p* < 0.001) (Figure 9).

There were no significant differences between the groups of two-piece or three-piece maxillary osteotomy. Surprisingly, the skeletal class did not influence the deviation from the virtual plan either.

We showed a positive correlation between the planned movement up/down to the deviation back/front (*p* = 0.021; correlation coefficient 0.202) and deviation in pitch (*p* < 0.01; correlation coefficient 0.376). There was a highly significant correlation between the planned pitch and the deviation in pitch (*p* < 0.001; correlation coefficient 0.489), planned right/left, and difference right/left (*p* < 0.001; correlation coefficient 0.335) as well as planned back/front to difference back/front (*p* < 0.001; correlation coefficient 0.345).

## 4. Discussion

This study aimed to quantify the accuracy of PSI-based osteosynthesis compared to conventional osteosynthesis with splint-based positioning in virtually planned segmented maxillary osteotomies during orthognathic surgery. In this collective, patient-specific implants significantly benefit an accurate translation of the virtual plan into the operative situs. 

Age, gender distribution, and the distribution of skeletal classes—with a class II dominance—represent a northern European collective [15]. We thereby conclude that the collective is an adequate representation of a standard patient collective.

Since the first description of the technique, segmental maxillary osteotomies have been in regular use but not in every department and in any form. This might be explained by the potential risks of segmental Le Fort I osteotomies that can occur, mainly when performed infrequently [10]. Considering that up to 30% of orthognathic patients show signs of a transversal deficit [16], the relatively rare use of the procedure is astonishing. Although there is evidence that segmental osteotomies are safe measures, a recent review has revealed that oral fistulas and damage to adjacent teeth are the most common complications. The most prevalent risk seems to be infection in up to 32% of all cases [17]. Centers with frequent use of the technique note no significant increase in risks through segmentation of the maxilla [18]. The most dreaded complication of all is the necrosis of the maxillary segments. There is good evidence that the perfusion of the mucosa is reduced after segmental Le Fort I osteotomies, but blood flow remains constant and equally distributed in all segments if conducted adequately [19]. Still, Lanigan described a series of 36 aseptic necroses, raising awareness for this most undesirable complication in an elective surgical procedure [20]. 

There are limited data regarding the accuracy of segmental maxillary Le Fort I osteotomies, especially under virtual planning. For unsegmented orthognathic surgery, an increasing number of publications have addressed the precision of the implementation with PSI. Diaconu has addressed this question in a systematic review and identifies a significantly higher accuracy of 0.85 mm and 2.35°, respectively, when comparing PSI to conventional osteosynthesis with 3D-printed splints or wafers (*p* < 0.01) [6]. The authors emphasize that literature is rare and especially heterogenic in methodology and quality regarding segmented Le Fort I procedures, mandibular osteotomies, or genioplasty. Greenberg quantified the discrepancy between PSI and conventional osteosynthesis at a mean of 1.129 mm [21]. In our study, the overall mean precision deficits of conventional osteosynthesis compared to patient-specific were transversally 0.1 mm, 1.93 mm vertically, and 1.30 mm anteriorly–posteriorly.

Regarding dimensional stability, there is even less evidence for unsegmented or segmented maxillary osteotomies. Two prospective studies have been conducted to address stability; no randomized controlled study has addressed the use of different types of osteosynthesis, to our knowledge [17]. In contrast to the differences in the precision of the surgical implementation of a plan, van der Wel could not describe a significant difference between conventional and patient-specific osteosynthesis when regarding postoperative stability [22]. 

The critical aspect of transversal expansion after three-piece maxillary osteotomies has been assessed by Strkrobo et al. [23]. Their retrospective cohort study showed a significant deficiency in posterior transversal widening of at least 0.7 mm (SD 0.83 mm) compared to the preoperative plan for the conventional technique. They found that a palatal extension of splints reduces the amount of failure. 

Our data support the assumption that there is a need for better posterior transversal development, as the lateral segments of two- or three-piece maxillae rotate posteriorly to the medial aspect, especially in conventional osteosynthesis. Still, PSI osteosynthesis shows the same problem, yet to a smaller extent.

In our analysis, the rotation centers of the segments are in the osteotomy gap; the reference point for our analysis is not in the center of the maxilla. The reason is that the fixed mucosa allows little translational movement in the alveolar crest. Therefore, the interpretation of the linear transversal deviation measurements must be relativized. To better describe the transversal widening of the maxilla in a segmental procedure, the rotation of the lateral segments on the z-axis (yaw) must be considered. An alternative to our measurement technique could have been the linear measurement of the anterior and posterior transversal width on the first premolar and first molar, as is often used in orthodontic plaster model analysis. However, these measurements cannot determine the segment position and precision of the implementation of the VSP. These dental landmarks are points, and the linear distance measurement between them cannot quantify the amount the alveolar ridge is dislocated.

Rios et al. published a case series with a comparable objective to our work. They conducted 22 consecutive multisegmented Le Fort I osteotomies and analyzed the position pre- and postoperatively. Nevertheless, their method is a landmark-based method that is insufficient in depicting the movements of whole segments. Manual landmark-based analysis can be prone to failure, as there is a high risk of cumulative error with every consecutive landmark identification. In their publication, the mean absolute discrepancies for the *x*-axis (transversal dimension), *y*-axis (anterior−posterior dimensions), and *z*-axis (vertical dimension) were 0.59 mm, 0.74 mm, and 0.56 mm [24], which is consistent with our findings. As an alternative to manual landmark identification, algorithmic approaches have been described as reducing the quantity of error [25]. Nevertheless, the author concluded that using PSI in segmental maxillary osteotomies is a safe measure, and the overall deviation from the plan was under 1 mm. Unfortunately, there was no control group to show conventional cases with the same method [24].

Tong Xi et al. described the advantages of voxel-based 3D analysis compared to landmark-based analysis, underlining the disadvantages of landmark-based analysis [26]. Meewis described a group of 67 patients with a regular Le Fort I osteotomy and ten multisegmental osteotomies. His two-dimensional measurements lacked a sufficient interpretation of the outcome [27].

There are few comparable studies surveying the precision of planning to implementation. Some authors use landmarks to describe the position of the segments, with the possibility of a cumulation of manually determining the points [28]. Kwon et al. [28] do not quantify the error of their method. They conclude that deviations of the segments were 0.96 ± 0.69 mm transversely, 1.23 ± 0.83 mm vertically, and 1.16 ± 0.80 mm anteroposterior. There is no explanation as to whether negative values have been considered absolute values; therefore, the interpretation of this work needs to be carefully weighed.

Although there is evidence that patient-specific implants help direct a plan with high precision, some authors find no significant advantage. Malenova et al. use five points to describe the position of the maxilla. Quantification of the individual or cumulative error was not elaborated, nor was the methodology scrutinized [29].

A comparable technique to our study was validated by Baan et al. in 2021. Their semiautomatic analyzing tool used similar matching techniques and showed an intra-class correlation of >0.92 and a low measurement variation (<0.673 ± 0.684 mm) [30].

The ICC is a tool that easily allows an assessment of the diagnostic quality of a method. The ICC of 0.86 in our collective is lower, but the mean absolute differences are under 0.5 mm and 0.5°, respectively.

Some aspects of virtual planning and implementation by guides and patient-specific implants are hard to put in numbers. In segmented maxillary cases, the option of virtually simulating the operation helps to a great extent. For instance, root angulation can be determined and cutting planes defined. Physical guides may help the surgeon in theatre to reduce the risk of dental injury. Furthermore, the almost automatic positioning of the bony segments into the individualized implant eases and accelerates the complex procedure. 

Still, these partially not-quantifiable benefits must be seen in relation to the high cost of patient-specific implants. Hanafy et al. [31] proved in a randomized controlled study that patient-specific implants are significantly more accurate. They, however, take the cost ratio into account and call the clinical benefits into question. The longer production time can be limiting, but scheduling surgery ten days after diagnostics in elective surgery is acceptable. Usually, the marking and drilling guides lead to extended surgical approaches, which might result in neurological disorders due to the greater wound. Smaller extensions and shorter drill guides can reduce this problem, such as paranasal minimal invasive PSI, as described by Swennen [12]. Nevertheless, they emphasize that the overall operation time can be reduced significantly, and even less experienced surgeons can achieve precise operation results [31]. In our collective, no deviation from the virtual plan forced us to discard a PSI. However, if the virtual plan is not conducted adequately or the PSI is not compliant, the individualized osteosynthesis allows no adaptation, and a switch to conventional osteosynthesis is needed.

It is safe to say that in any orthognathic case, patient-specific osteosynthesis can be beneficial. But when to indicate the use of it is still a very vaguely defined corridor. Kreaima et al. suggested using PSI in cases exceeding 3.7 mm anteroposterior translations in regular Le-Fort I osteotomies [32]. These data are in congruence with our findings, primarily due to the amount of dislocation, which positively correlates with the planned movement. Secondarily, splint-based surgery in our collective did not reach positions as far anteriorly as virtually planned.

## 5. Conclusions

PSI is an osteosynthesis form that helps conduct a virtual surgical plan significantly more precisely than conventional osteosynthesis in segmental maxillary procedures. Yet in times of limited resources, PSI should be indicated where it seems most beneficial. It is, therefore, adequate to discuss the use in simple, unsegmented orthognathic cases critically. In our view, the number of segments and the extent of the planned movement, as seen in severe transversal maxillary deficit or asymmetrical cases, can be considered when deciding to opt for patient-specific osteosynthesis. Further development of PSI designs might increase the precision of segmental maxillary osteotomies.

## Figures and Tables

**Table 1:** List of all measured translations: mean values and standard deviation (sd) in mm. Frontal segments only in case of a three-piece maxilla. Lateral segments are shown for both segmentation techniques.

	Deviation from the Virtual Plan in mm		
Osteosynthesis	Right(−)/Left(+)	Back(−)/Front(+)	Down(−)/Up(+)
PSI (frontal segments)	−0.015 (sd 0.7641)	−0.4889 (sd 1.49531)	−1.2017 (sd 1.34432)
Conv. (frontal segments)	−0.63 (sd 1.78276)	−2.0458 (sd 2.30621)	−2.43 (sd 1.63363)
PSI (right segments)	−0.0811 (sd 1.36651)	−0.6611 (sd 1.62181)	−1.5557 (sd 1.84554)
Conv. (right segments)	−0.2523 (sd 2.52552)	−1.3564 (sd 2.66733)	−3.22 (sd 3.52955)
PSI (left segments)	−0.764 (sd 0.868)	−0.6921 (sd 1.75916)	−1.3482 (sd 1.50505)
Conv. (left segments)	0.1695 (sd 2.06619)	−0.845 (sd 2.55035)	−2.3168 (sd 3.30965)

**Table 2:** List of all the measured rotations for the individual segments: mean values and standard deviation (sd) in degrees °.

Deviation from the Virtual Plan in Degrees °			
Osteosynthesis	Roll	Pitch	Yaw
PSI (frontal segments)	0.2806 (sd 1.11487°)	0.1244 (sd 2.85313°)	−0.1944 (sd 2.50673°)
Conv. (frontal segments)	1.355 (sd 2.14127°)	1.5558 (sd 3.62935°)	−2.1217 (sd 2.25613°)
PSI (right segments)	−0.0468 (sd 2.61731°)	0.9175 (sd 2.33728°)	−0.16 (sd 1.75085°)
Conv. (right segments)	−0.0532 (sd 3.56789°)	2.98 (sd 4.10364°)	−1.42 (sd 3.12374°)
PSI (left segments)	0.1261 (sd 2.71897°)	0.6886 (sd 2.71551°)	0.6275 (sd 1.96414°)
Conv. (left segments)	2.0786 (sd 5.26521°)	3.5732 (sd 4.27475°)	1.2982 (sd 2.76101°)

## Data Availability

The collected data may be shared upon request from the first author.

## References

[1] Jandali D., Barrera J.E. (2020). Recent advances in orthognathic surgery. Curr. Opin. Otolaryngol. Head Neck Surg..

[2] Alkhayer A., Piffko J., Lippold C., Segatto E. (2020). Accuracy of virtual planning in orthognathic surgery: A systematic review. Head Face Med..

[3] Tondin G.M., Leal M., Costa S.T., Grillo R., Jodas C.R.P., Teixeira R.G. (2022). Evaluation of the accuracy of virtual planning in bimaxillary orthognathic surgery: A systematic review. Br. J. Oral Maxillofac. Surg..

[4] Ruckschloss T., Ristow O., Muller M., Kuhle R., Zingler S., Engel M., Hoffmann J., Freudlsperger C. (2019). Accuracy of patient-specific implants and additive-manufactured surgical splints in orthognathic surgery—A three-dimensional retrospective study. J. Craniomaxillofac. Surg..

[5] Ruckschloss T., Ristow O., Kuhle R., Weichel F., Roser C., Aurin K., Engel M., Hoffmann J., Freudlsperger C. (2020). Accuracy of laser-melted patient-specific implants in genioplasty—A three-dimensional retrospective study. J. Craniomaxillofac. Surg..

[6] Diaconu A., Holte M.B., Berg-Beckhoff G., Pinholt E.M. (2023). Three-Dimensional Accuracy and Stability of Personalized Implants in Orthognathic Surgery: A Systematic Review and a Meta-Analysis. J. Pers. Med..

[7] Li B., Wei H., Jiang T., Qian Y., Zhang T., Yu H., Zhang L., Wang X. (2021). Randomized Clinical Trial of the Accuracy of Patient-Specific Implants versus CAD/CAM Splints in Orthognathic Surgery. Plast Reconstr. Surg..

[8] Steinhauser E.W. (1972). Midline splitting of the maxilla for correction of malocclusion. J. Oral Surg..

[9] Turvey T.A. (1985). Maxillary expansion: A surgical technique based on surgical-orthodontic treatment objectives and anatomical considerations. J. Maxillofac. Surg..

[10] Posnick J.C., Adachie A., Choi E. (2016). Segmental Maxillary Osteotomies in Conjunction with Bimaxillary Orthognathic Surgery: Indications—Safety—Outcome. J. Oral Maxillofac. Surg..

[11] De Waard O., Baan F., Verhamme L., Breuning H., Kuijpers-Jagtman A.M., Maal T. (2016). A novel method for fusion of intra-oral scans and cone-beam computed tomography scans for orthognathic surgery planning. J. Craniomaxillofac. Surg..

[12] Swennen G.R., Mollemans W., Schutyser F. (2009). Three-dimensional treatment planning of orthognathic surgery in the era of virtual imaging. J. Oral Maxillofac. Surg..

[13] Maes F., Collignon A., Vandermeulen D., Marchal G., Suetens P. (1997). Multimodality image registration by maximization of mutual information. IEEE Trans. Med. Imaging.

[14] De Groeve P., Schutyser F., Van Cleynenbreugel J., Suetens P. (2001). Registration of 3D Photographs with Spiral CT Images for Soft Tissue Simulation in Maxillofacial Surgery.

[15] Alhammadi M.S., Halboub E., Fayed M.S., Labib A., El-Saaidi C. (2018). Global distribution of malocclusion traits: A systematic review. Dental Press J. Orthod..

[16] Proffit W.R., Phillips C., Dann C.t. (1990). Who seeks surgical-orthodontic treatment?. Int. J. Adult Orthod. Orthognath. Surg..

[17] Haas Junior O.L., Guijarro-Martinez R., de Sousa Gil A.P., da Silva Meirelles L., de Oliveira R.B., Hernandez-Alfaro F. (2017). Stability and surgical complications in segmental Le Fort I osteotomy: A systematic review. Int. J. Oral Maxillofac. Surg..

[18] Joseph M.M., Jain N.S., DeLong M.R., Ozaki W. (2023). Association between Maxillary Segmentation and Perioperative Complications in Le Fort I Osteotomy. J. Craniofac. Surg..

[19] Kretschmer W.B., Baciut G., Baciut M., Zoder W., Wangerin K. (2009). Changes in bone blood flow in segmental LeFort I osteotomies. Oral Surg. Oral Med. Oral Pathol. Oral Radiol. Endod..

[20] Lanigan D.T., Hey J.H., West R.A. (1990). Aseptic necrosis following maxillary osteotomies: Report of 36 cases. J. Oral Maxillofac. Surg..

[21] Greenberg S., Buchbinder D., Turner M.D., Dhillon P., Afshar A.A. (2021). Three-Dimensional Repositioning of the Maxilla in Orthognathic Surgery Using Patient-Specific Titanium Plates: A Case Series. J. Oral Maxillofac. Surg..

[22] Van der Wel H., Kraeima J., Spijkervet F.K.L., Schepers R.H., Jansma J. (2023). Postoperative skeletal stability at the one-year follow-up after splintless Le Fort I osteotomy using patient-specific osteosynthesis versus conventional osteosynthesis: A randomized controlled trial. Int. J. Oral Maxillofac. Surg..

[23] Stokbro K., Aagaard E., Torkov P., Marcussen L., Bell R.B., Thygesen T. (2017). Surgical Splint Design Influences Transverse Expansion in Segmental Maxillary Osteotomies. J. Oral Maxillofac. Surg..

[24] Rios O., Lerhe B., Chamorey E., Savoldelli C. (2022). Accuracy of Segmented Le Fort I Osteotomy with Virtual Planning in Orthognathic Surgery Using Patient-Specific Implants: A Case Series. J. Clin. Med..

[25] Gupta A., Kharbanda O.P., Sardana V., Balachandran R., Sardana H.K. (2015). A knowledge-based algorithm for automatic detection of cephalometric landmarks on CBCT images. Int. J. Comput. Assist. Radiol. Surg..

[26] Xi T., van Luijn R., Baan F., Schreurs R., de Koning M., Berge S., Maal T. (2020). Landmark-Based Versus Voxel-Based 3-Dimensional Quantitative Analysis of Bimaxillary Osteotomies: A Comparative Study. J. Oral Maxillofac. Surg..

[27] Meewis J., Govaerts D., Falter B., Grisar K., Shaheen E., Van de Vyvere G., Politis C. (2018). Reaching the vertical versus horizontal target position in multi-segmental Le Fort I osteotomy is more difficult, but yields comparably stable results to one-segment osteotomy. Int. J. Oral Maxillofac. Surg..

[28] Kwon T.G., Miloro M., Han M.D. (2020). How Accurate Is 3-Dimensional Computer-Assisted Planning for Segmental Maxillary Surgery?. J. Oral Maxillofac. Surg..

[29] Malenova Y., Ortner F., Liokatis P., Haidari S., Troltzsch M., Fegg F., Obermeier K.T., Hartung J.T., Kakoschke T.K., Burian E. (2023). Accuracy of maxillary positioning using computer-designed and manufactured occlusal splints or patient-specific implants in orthognathic surgery. Clin. Oral Investig..

[30] Baan F., Sabelis J.F., Schreurs R., van de Steeg G., Xi T., van Riet T.C.T., Becking A.G., Maal T.J.J. (2021). Validation of the OrthoGnathicAnalyser 2.0-3D accuracy assessment tool for bimaxillary surgery and genioplasty. PLoS ONE.

[31] Hanafy M., Akoush Y., Abou-ElFetouh A., Mounir R.M. (2020). Precision of orthognathic digital plan transfer using patient-specific cutting guides and osteosynthesis versus mixed analogue-digitally planned surgery: A randomized controlled clinical trial. Int. J. Oral Maxillofac. Surg..

[32] Kraeima J., Schepers R.H., Spijkervet F.K.L., Maal T.J.J., Baan F., Witjes M.J.H., Jansma J. (2020). Splintless surgery using patient-specific osteosynthesis in Le Fort I osteotomies: A randomized controlled multi-centre trial. Int. J. Oral Maxillofac. Surg..

